# The evolutionary neuroscience of musical beat perception: the Action Simulation for Auditory Prediction (ASAP) hypothesis

**DOI:** 10.3389/fnsys.2014.00057

**Published:** 2014-05-13

**Authors:** Aniruddh D. Patel, John R. Iversen

**Affiliations:** ^1^Department of Psychology, Tufts UniversityMedford, MA, USA; ^2^Swartz Center for Computational Neuroscience, Institute for Neural Computation, University of California San DiegoLa Jolla, CA, USA

**Keywords:** evolution, rhythm perception, brain, music cognition, comparative psychology

## Abstract

Every human culture has some form of music with a beat: a perceived periodic pulse that structures the perception of musical rhythm and which serves as a framework for synchronized movement to music. What are the neural mechanisms of musical beat perception, and how did they evolve? One view, which dates back to Darwin and implicitly informs some current models of beat perception, is that the relevant neural mechanisms are relatively general and are widespread among animal species. On the basis of recent neural and cross-species data on musical beat processing, this paper argues for a different view. Here we argue that beat perception is a complex brain function involving temporally-precise communication between auditory regions and motor planning regions of the cortex (even in the absence of overt movement). More specifically, we propose that simulation of periodic movement in motor planning regions provides a neural signal that helps the auditory system predict the timing of upcoming beats. This “action simulation for auditory prediction” (ASAP) hypothesis leads to testable predictions. We further suggest that ASAP relies on dorsal auditory pathway connections between auditory regions and motor planning regions via the parietal cortex, and suggest that these connections may be stronger in humans than in non-human primates due to the evolution of vocal learning in our lineage. This suggestion motivates cross-species research to determine which species are capable of human-like beat perception, i.e., beat perception that involves accurate temporal prediction of beat times across a fairly broad range of tempi.

## Introduction

Music exists in every human culture, and every culture has some form of music with a beat: a perceived periodic pulse that listeners use to guide their movements and performers use to coordinate their actions (Nettl, 2000; Brown and Jordania, 2013). What brain mechanisms support beat perception, and how did these mechanisms evolve? One possibility is that the relevant neural mechanisms are very ancient. This is an intuitively appealing view, as rhythm is often considered the most basic aspect of music, and is increasingly thought to be a fundamental organizing principle of brain function (Buzsáki, 2006). The view is also consonant with Darwin's ideas about the evolution of human musicality. Darwin believed that our capacity for music had deep evolutionary roots and argued that “The perception, if not the enjoyment, of musical cadences and of rhythm is probably common to all animals, and no doubt depends on the common physiological nature of their nervous systems” (Darwin, 1871).

This view has been echoed by several modern researchers. For example, Hulse et al. (1995) argues that “There is increasing evidence that some of the principles governing human music perception and cognition may also hold for non-human animals, such as the perception of tempo and rhythm.” More recently, Large and colleagues (e.g., Large, 2008; Large and Snyder, 2009) have proposed a theory of musical beat perception based on very general neural mechanisms, building on the dynamic attending theory of Jones (e.g., Jones and Boltz, 1989; Large and Jones, 1999). According to this “neural resonance” theory, beat perception arises when non-linear oscillations in the nervous system entrain to (oscillate in synchrony with) external rhythmic stimuli. As stated by Large and Snyder (2009), “Non-linear oscillations are ubiquitous in brain dynamics and the theory asserts that some neural oscillations -perhaps in distributed cortical and subcortical areas - entrain to the rhythms of auditory sequences.” Large's ideas are in line with Darwin's views because neural resonance theory “holds that listeners experience dynamic temporal patterns (i.e., pulse and meter) … because they are intrinsic to the physics of the neural systems involved in perceiving, attending, and responding to auditory stimuli.” Neural resonance theory is interesting in light of other mechanistic proposals for the interaction of attention, neural oscillators, and the temporal dynamics of sensory signals in the brain (Schroeder and Lakatos, 2009).

There are, however, reasons to suggest that entrainment of auditory neural activity to external rhythms is not sufficient to explain beat perception. One such reason is that “pure perception” of a musical beat (i.e., listening in the absence of overt movement) strongly engages the motor system, including regions such as premotor cortex, basal ganglia, and supplementary motor regions (Chen et al., 2008a; Grahn and Rowe, 2009; Kung et al., 2013). In other words, there is an intimate connection between beat perception and motor functions of the brain, and any theory of beat perception needs to account for this coupling. Second, recent EEG work on rhesus monkeys (*Macaca mulatta*) suggests that they do not perceive a beat in rhythmic auditory patterns (Honing et al., 2012). This EEG study followed earlier work showing that monkeys could not learn to tap in synchrony with an auditory (or a visual) metronome, a task which is trivially easy for humans, even for those with no musical training (Zarco et al., 2009). This was the first study to train monkeys (or for that matter, any animal) to move in synchrony with a metronome, a task that has been extensively studied in human cognitive science (Repp and Su, 2013). The study produced several surprising results. While the monkeys could successfully listen to two metronome clicks and then reproduce the same interval by tapping twice on a key, they had great difficulty learning to tap in synchrony with a metronome of several beats. Specifically, each monkey took over a year of training to learn the metronome task, and when tested, their taps were always a few 100 ms after each metronome click rather than aligned with it. This is quite unlike humans: when humans are asked to tap with a metronome, they spontaneously align their taps closely in time with metronome clicks (i.e., within a few tens of ms). This human tendency for “phase alignment” between taps and beats indicates that humans accurately predict the timing of upcoming beats. In contrast, monkey rhythmic tapping did not show this sort of predictive behavior. To be sure, the monkeys did show shorter tapping latencies to metronomic vs. irregularly-timed clicks, suggesting they had some predictive capacities. Furthermore, monkey and human tapping to a metronome both showed the scalar property of temporal processing, whereby temporal variability between taps scaled with interval duration. What was striking, however, was the lack of phase alignment between taps and metronome events in monkeys.

This inability to accurately align movement with discrete, periodic events is particularly surprising given that monkey motor cortex can represent time-to-contact in a predictive manner when doing an interception task involving a continuously-moving visual object (Merchant et al., 2004; Merchant and Georgopoulos, 2006). Recently, based on the results of Zarco et al. (2009) and subsequent studies, including studies which characterize the neurophysiological properties of cells in medial premotor areas and the putamen during synchronization-continuation tapping tasks (e.g., Merchant et al., 2011, 2013a,b; Bartolo et al., 2014), Merchant and Honing (2014) have proposed that monkeys and humans share neural mechanisms for interval-based timing (i.e., timing of single intervals), but may differ in the mechanisms involved in beat-based timing.

The above research with humans (showing extensive activation of the motor system in pure beat perception) and with monkeys (suggesting that they may lack human-like beat perception) suggests that entrainment of auditory cortical activity to external rhythms is not a sufficient explanation of beat perception. Here we advance a view of musical beat perception which can account for auditory-motor interactions in pure perception of a beat, and which can also account for species-restrictedness in the capacity for beat perception. In terms of auditory-motor interactions, we argue that musical beat perception (even in the absence of overt movement) relies on a simulation of periodic action in motor planning regions of the brain, and on bidirectional signaling between these regions and auditory regions. In terms of species-restrictedness, we suggest that only some species may have the requisite neural connections to support these specific auditory-motor interactions.

The paper is organized into three sections. The first section discusses some key aspects of musical beat perception, including the predictive and flexible nature of beat perception. The second section focuses on the brain's ability to predict the timing of beats, introduces the “action simulation for auditory prediction” (ASAP) hypothesis, and discusses three testable predictions made by this hypothesis. The third section discusses possible neural substrates for auditory-motor interactions in beat perception, and suggests why the relevant neural pathways may be restricted to certain species. It should be emphasized at the outset that the ASAP hypothesis and the species-restrictedness of beat perception are conceptually distinct ideas. That is, the ASAP hypothesis does not *require* the assumption that beat perception is species-restricted, although this paper links these ideas together. It is also worth noting that the ASAP hypothesis, while involving the idea of motor simulation, does not involve the mirror neuron system (a point further discussed in the section on possible neural substrates).

## Some key aspects of human musical beat perception

### Beat perception is predictive

Musical beat perception involves perceiving a periodic pulse in spectotemporally complex sound sequences. Listeners often express their perception of the pulse by moving rhythmically in synchrony with the pulse, e.g., via head bobbing, foot tapping, or dance. (Informally, the beat is what we tap our foot to when listening to music. In the laboratory, this rhythmic response to music can easily be studied by asking people to tap a finger to the perceived beat, e.g., Iversen and Patel, 2008). The manner in which people synchronize to the beat reveals that musical beat perception is a predictive process. Specifically, taps fall very close to beats in time (i.e., within a few tens of ms of beats) showing that the brain makes highly accurate temporal predictions about the timing of upcoming beats (Rankin et al., 2009; for further evidence of the anticipatory nature of movement to a beat see Van der Steen and Keller, 2013).

Accurate temporal prediction of beat times has consequences for perception even in the absence of movement. Several studies have shown facilitated perceptual processing of auditory events which occur on (vs. off) the beat (Escoffier et al., 2010; Geiser et al., 2012). This body of findings is consistent with Jones's “Dynamic Attending Theory” (Jones and Boltz, 1989), which posits an increase of “attentional energy” at expected times of the beat and focuses perceptual processing resources on those times. This temporal facilitation even extends to the processing of non-auditory events. For example, (Escoffier et al., 2010) showed facilitation of visual image processing when images occurred on (vs. off) the beat of an accompanying auditory pattern. More generally, it appears that the prediction of auditory beats has broader cognitive consequences, including facilitating the learning and recall of strongly beat-inducing rhythmic patterns (Povel and Essens, 1985).

### Beat perception is flexible across a wide range of tempi

Humans can perceive musical beats across a wide range of tempi. We perceive beats in a range of about 250 ms–2 s, though intervals between about 400 and 1200 ms give rise to the strongest sense of beat, and humans show a preference for beat periods around 600 ms (London, 2012). In dance music (i.e., music designed to convey a clear sense of a beat), pieces tend to have tempi between 94 and 176 beats per minute (BPM) (van Noorden and Moelants, 1999). Within this range, van Noorden and Moelants (1999) found a preponderance of pieces between 120 and 150 BPM, and a median tempo of 133 BPM, corresponding to one beat every 451 ms. Given this median tempo, it appears that humans can easily synchronize to beats which are about 30% slower than this tempo (i.e., 94 BPM) or about 30% faster than this tempo (i.e., 176 BPM). This tempo flexibility of beat perception and synchronization can be contrasted with many other examples of synchrony in nature, such as the synchronous chirping of certain cricket species or the synchronous flashing of certain firefly species, which is limited to a rather narrow tempo range (e.g., for fireflies, ±10% relative to the spontaneous flash rate, cf. Figure 2 of Hanson et al., 1971).

### Beat perception is constructive

**Figure 1:**
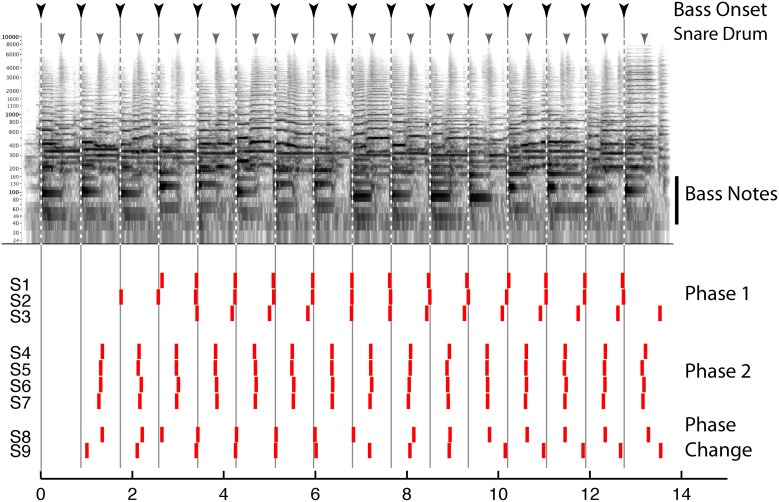
**Top:** Spectrogram of an excerpt of jazz music (“Stompin at the Savoy,” by Benny Goodman; for corresponding audio, see supplementary sound file 1). Inverted arrows above the spectrogam show times of double bass and snare drum onsets, respectively. **Bottom**: time at which 9 human subjects (S1–9) tapped when instructed to “tap to the beat you hear.” Each tap is indicated by a vertical red bar. See text for details.

Behavioral evidence suggests that beat perception involves more than the passive entrainment of neural responses to sound. This evidence concerns the fact that the beat imposed on a given sound can be consciously altered by the listener, and this manipulation can radically reshape how that sound is heard Thus, beat perception is not merely the “discovery” of periodicity in complex sounds, but is more active and under voluntary control, and provides an internal temporal reference that shapes rhythm perception. For example, the beat guides attention in time, influences accent perception, and determines grouping boundaries between rhythmic patterns (Repp, 2007; Locke, 2009). While much popular music is composed in such a way as to guide the listeners' beat perception (e.g., by physically accenting the beats or emphasizing them with grouping boundaries, instrumentation, or melodic contours), music with weaker cues may be more ambiguous and can lead to multiple interpretations of the beat. These can include interpretations with little support from the stimulus (e.g., as marked by the coincidence of notes with the beat). Such multiplicity of beat interpretations is demonstrated in Figure 1, which shows how different listeners' responses can be when instructed to “tap to the beat you hear” in an excerpt of jazz as part of the “Beat Alignment Test” (BAT) for the assessment of beat production and perception (Iversen and Patel, 2008). The data emphasize that the acoustic signal does not determine the beat: individuals picked different phases for their taps, corresponding to taps on the downbeat with the bass note (Phase 1), or on the upbeat with the snare drum (Phase 2). Listeners can also shift their beat phase midstream (S8 and 9).

Such phase flexibility was studied by Repp et al. (2008) who showed that listeners could synchronize with rhythmic sequences successfully both at the beat phase most strongly supported by the stimulus, but also at other phases that had little acoustic support and which corresponded to highly syncopated rhythms. The ability to maintain a beat that conflicts with the acoustic signal is strong evidence for the constructed nature of the beat, and the ability to voluntarily shift the phase of the internal beat relative to the stimulus has been exploited by neuroscientific experiments discussed below (Iversen et al., 2009).

Importantly, a listener's placement of the beat has a profound influence on their perception of temporal patterns (Repp et al., 2008). That is, identical temporal patterns of notes heard with different beat interpretations can sound like completely different rhythms to listeners (Repp, 2007; Iversen et al., 2009), indicating the influence of beat perception on rhythm perception more generally. Thus, the beat seems to serve as a temporal scaffold for the encoding of patterns of time, and rhythm perception depends not only on the stimulus but on the timing of the endogenous sense of beat.

### Beat perception is hierarchical

Beats are often arranged in patterns that create higher-level periodicities, for example a “strong” beat every 2 beats (which creates a march-like pattern) or every three beats (which creates a waltz-like pattern). This hierarchical patterning of beats is referred to as meter. When asked to “tap to the beat of music,” an individual listener can often switch between which metric level she or he synchronizes with. Audio examples are provided in supplementary sound files 2 and 3: sound file 2 presents a simple Western melody, while sound file 3 presents this melody twice, with “tapping” at different metrical levels [taps are indicated by percussive sounds]. The notation of this melody and a metrical grid showing the different hierarchical levels of beats can be found in Chapter 3 of Patel (2008). Numerous studies have found that listeners tend to pick the level of the hierarchy closest to the human preferred tempo range of about 600 ms between beats (see above), but there is considerable individual variation, with some listeners picking metrical levels either faster and slower than this (Drake et al., 2000; Toiviainen and Snyder, 2003; McKinney and Moelants, 2006; Martens, 2011).

### Beat perception is modality-biased

Rhythmic information can be transmitted to the brain via different modalities, e.g., via auditory vs. visual signals. Yet in humans the same rhythmic patterns can give rise to a clear sense of a beat when presented as sequences of tones but not when presented as sequences of flashing lights (Patel et al., 2005; McAuley and Henry, 2010; Grahn et al., 2011, but see Iversen et al., in press; Grahn, 2012, for evidence that *moving* visual stimuli may give rise to a sense of beat). This may be one reason why humans synchronize so much better with auditory vs. visual metronomes, even when they have identical timing characteristics (e.g., Chen et al., 2002; Repp and Penel, 2002; Hove et al., 2010; Iversen et al., in press). Interestingly, when monkeys tap with a metronome, they do not synchronize any better with auditory than with visual metronomes, and in fact find it easier to learn to tap with a visual metronome (Zarco et al., 2009; Merchant and Honing, 2014).

### Beat perception engages the motor system

An important finding in the neuroscience of beat perception is that pure perception of a beat (i.e., in the absence of any overt movement) engages motor areas of the brain, including premotor cortex (PMC), the basal ganglia (putamen), and supplementary motor area (SMA) (e.g., Grahn and Brett, 2007; Chen et al., 2008a; Grahn and Rowe, 2009; Geiser et al., 2012; Teki et al., 2012; Kung et al., 2013). Beat perception in auditory rhythms is also associated with enhanced functional coupling between auditory and motor regions (Kung et al., 2013), and this coupling appears to be stronger in musicians than in non-musicians (Grahn and Rowe, 2009). Grahn and Rowe (2009) have suggested that a cortico-subcortical network including the putamen, SMA, and PMC is engaged in the analysis of temporal sequences and prediction or generation of putative beats (cf. Teki et al., 2012). Zatorre et al. (2007) have suggested that auditory-premotor interactions in particular underlie the temporal predictions involved in rhythm perception. More generally, a role for the motor system in prediction of events in structured sequences has been proposed by Schubotz (2007). Going even further, Rauschecker and Scott (2009) have suggested that the premotor cortex (and associated structures of the dorsal auditory stream) have evolved primarily for the purpose of timing in sequences, a function used both by the motor system in programming motor sequences and by the auditory system in predicting the structure of acoustic sequences (cf. Leaver et al., 2009 for relevant fMRI data). These ideas provide a foundation for the current work, which seeks to explain why and how the motor system is involved in predicting the timing of auditory beats, and why this ability may be restricted to certain species.

## The action simulation for auditory prediction (ASAP) hypothesis

### Overview of the hypothesis

The ASAP hypothesis suggests that the motor planning system uses a simulation of body movement (specifically, of periodic movement patterns) to entrain its neural activity patterns to the beat period, and that these patterns are communicated from motor planning regions to auditory regions where they serve as a predictive signal for the timing of upcoming beats and shape the perceptual interpretation of rhythms. This hypothesis expands on an earlier hypothesis proposed by Iversen et al. (2009) that in beat perception the motor system affects the auditory system by setting up precisely-timed beat related expectations. The current section focuses on cognitive aspects of this hypothesis: discussion of specific neural substrates is deferred to the following section (“Neural substrates for auditory-motor interactions in beat perception: an evolutionary perspective”). For the purposes of the current section, “motor planning regions” should be taken as a functional label for a collection of regions including PMC, SMA, putamen, and other motor regions which have been shown to be active in fMRI studies of pure beat perception.

Why would the auditory system become functionally coupled to motor planning regions in order to make predictions about the timing of auditory events? (Recall that the focus here is on pure perception of a beat, not on synchronized movement to a beat.) We suggest that it is the *periodic* nature of musical beats, and the timescale of their occurrence (typically on the order of several 100 ms between beats) that leads the auditory system to couple with the motor planning system as a resource for making temporal predictions. The motor system is an excellent generator of neural periodicities in this time range, because humans frequently make periodic motions at the time scale of several 100 ms, including intervals between footfalls when walking, or between arm swings or pulls when pounding or pulling (Styns et al., 2007). Hence an internal simulation of periodic motion (decoupled from actual movement) may be one way for the brain to generate neural signals that can be used to make temporal predictions about discretely-timed periodic auditory events. The internal simulation may be at an abstract level, not tied to a specific effector, and need not be related to motor imagery (Schubotz, 2007).

It is worth noting that the ASAP hypothesis bears a broad architectural similarity to a mechanism proposed to account for superior encoding and long-term retention of auditory stimuli in humans when compared to non-human primates (Schulze et al., 2012). This view hypothesizes that orofacial articulatory motor regions are essential to the effective encoding of fast chains of auditory stimuli in humans in a way that enables their long-term retention after single exposures. Such a combined auditory/motor representation is argued to be more easily stored than a purely auditory representation. This hypothesis differs from ASAP in that ASAP posits the role of the motor system is in temporal prediction and in modifying ongoing perception, rather than in encoding sounds in memory. However, both hypotheses suggest that the motor system is recruited for auditory perception because of its unique temporal properties, and that auditory perception involves motor signals returning to the auditory system.

### ASAP and movement to the beat of music

One appeal of the ASAP hypothesis is that it suggests a natural explanation for why we move rhythmically to the beat of music in the first place, and why such movements tend to be predictive rather than reactive. If the motor planning system is used to predict the timing of beats via a simulation of periodic movement, then actual periodic movements to music are a natural consequence of this arrangement: they emerge when activity in the motor planning regions is allowed to influence nearby brain regions which directly control movement (e.g., primary motor cortex). Furthermore, if the motor planning system predicts the timing of upcoming beats, then real movements to musical rhythms should be predictive rather than reactive, which is what is typically observed. The ASAP hypothesis also suggests an explanation for why humans move rhythmically to music using complex, multi-timescale movements (Toiviainen et al., 2010; see also video examples in Burger et al., 2013). Specifically, since beat perception in music is hierarchical (as discussed in the previous section), the motor system may make predictions about beat timing at different hierarchical timescales by associating different timescales with simulations of periodic movement in different motor effectors (e.g., with hand/arm movements at twice the rate of simultaneous step-like movements, cf. Toiviainen et al., 2010).

The ASAP hypothesis is also relevant to the question of why moving to the beat of music is pleasurable (cf. Zatorre and Salimpoor, 2013). Prominent theories of music cognition have long postulated in an intimate relationship between expectation and emotion in music perception (e.g., Meyer, 1956; Huron, 2006). According to this view, which has inspired a good deal of empirical research (e.g., Steinbeis et al., 2006), music perception is a form of “predictive listening” in which listeners have expectations about upcoming events and the confirmation or denial of these expectations arouses emotion. Beat perception is a predictive process, and when people move in synchrony with a beat, the close alignment of movements with beats provides the brain with evidence that temporal predictions were accurate. According to Zald and Zatorre (2012), “prediction confirmation” is rewarding to the brain. Hence prediction confirmation could be one reason why moving in synchrony with the beat is pleasurable to humans. Furthermore, if movements (and the predictions behind them) are hierarchical, as alluded to in the previous paragraph, then simultaneously moving different effectors at different timescales may provide more rewards than moving at just one timescale.

### Neural data consistent with ASAP

From a neuroscience perspective, the most important claim of the ASAP hypothesis is that beat perception involves temporally precise two-way communication between auditory regions and motor planning regions. This is related to the concept of reentry, “a process of temporally ongoing parallel signaling between separate maps along ordered anatomical connections” (Edelman, 1989, p. 65). According to ASAP, (1) neural signals go from auditory to motor planning regions to provide information about the timing of auditory events; (2) these signals influence the timing of periodic motor planning signals in motor regions, and (3) these planning signals go from motor regions back to auditory regions to provide a signal that predicts upcoming beat times. According to this view, one component of beat perception is periodic reentrant *input* from motor planning regions into auditory cortex, with this input being precisely timed around the location of auditory beats. This idea of perception involving input from motor to auditory cortex resonates with the “inverse model” idea articulated in Rauschecker and Scott (2009), Rauschecker (2011), and other studies.

**Figure 2:**
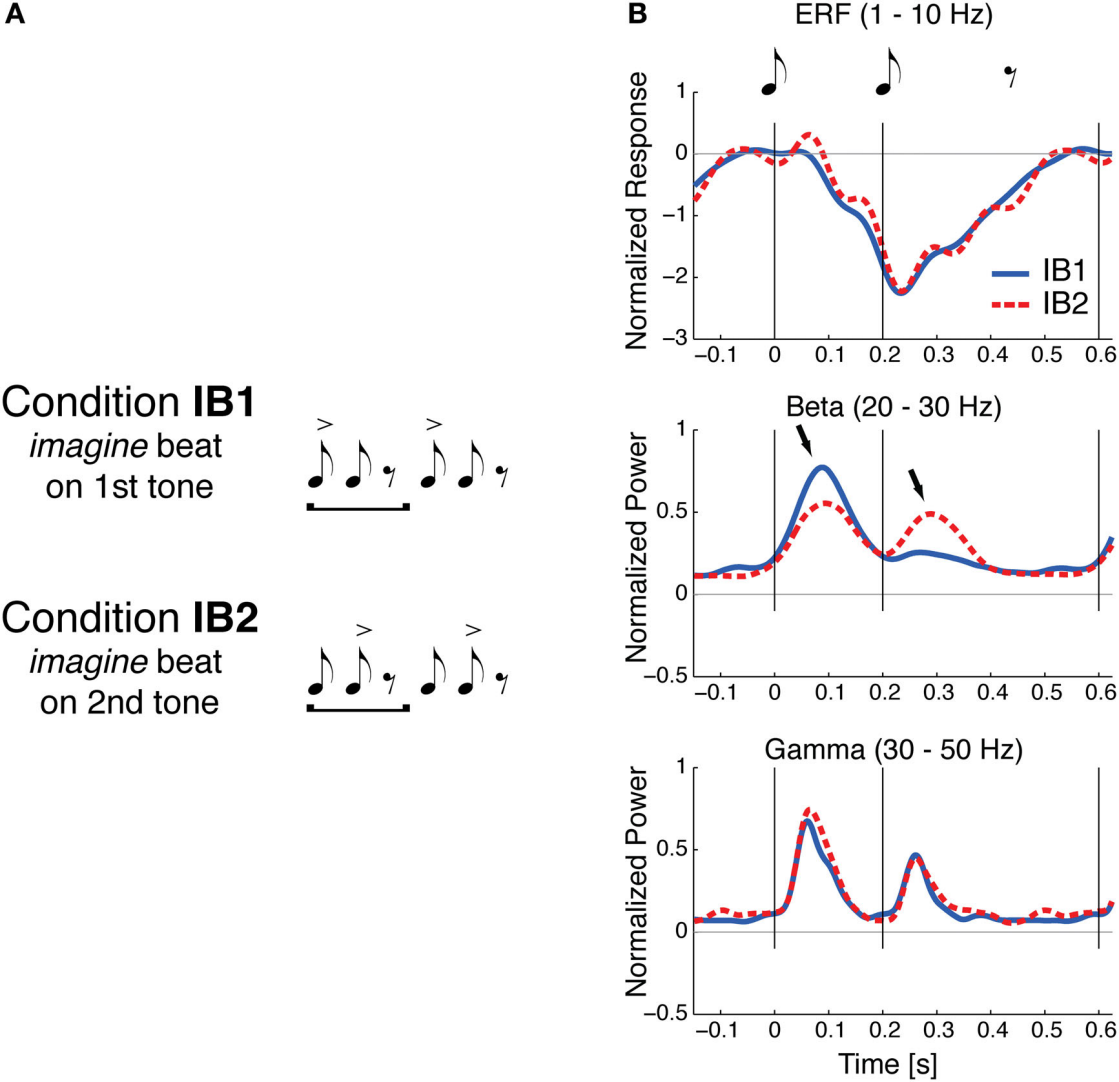
**Sound evoked responses are modulated by beat perception, from Iversen et al. (2009). (A)** Illustration of two conditions in the study of Iversen et al. (2009). A simple two-note repeating pattern is heard by listeners. On some trials listeners imagine the beat is on the first tone (condition IB1); on other trials, they imagine the beat is on the 2nd tone (condition IB2). The accents indicate the *imagined* beat and do not correspond to any physical differences in the stimulus, which were identical in the two conditions. **(B)** Normalized evoked neural responses for the two imagined beat conditions, measured with MEG. Thin gray vertical lines at 0 and 200 ms indicate onset of the two tones (each 45 ms long and 1 KHz in frequency). Solid blue line: evoked response when beat was imagined on tone 1; dashed red line: evoked response when beat was imagined on tone 2. Grand averages are shown for three frequency bands: Event-related field ERF (1–10 Hz), beta (20–30 Hz), and gamma (30–50 Hz). For beta and gamma frequencies, the mean power envelopes were averaged across individuals after first normalizing each individual's peak power across both conditions to one. Statistically significant effects of imagined beat location occurred only in the beta frequency response, where the response to both tones 1 and 2 was larger when that tone was imagined to be on the beat (arrows).

If beat perception involves periodic input from motor planning regions into auditory regions, one might expect early auditory responses to events perceived as “on the beat” to be distinct from responses to similar events not on the beat. Using magnetoencephalograpy (MEG), Iversen et al. (2009) found evidence for this phenomenon. Participants listened (without moving) to a simple repeating pattern of two tone pips (with an interonset interval of 200 ms) followed by a silence of 400 ms. On alternate trials listeners imagined that either the first or second note was on the beat (Figure 2A). That is, beat perception was manipulated without changing the auditory stimulus. When a tone was on the imagined beat, larger evoked neural responses to the tone were observed in the upper beta frequency range (20–30 Hz), but not in other frequency ranges, e.g., in the 1–10 Hz range typically analyzed for event-related potentials or in the 30–50 Hz gamma range (Figure 2B). This pattern of neural response contrasted with a control condition in which events on the beat were physically accented via an intensity boost: in that case larger neural responses to on-beat tones were observed across the three frequency ranges.

Iversen et al.'s (2009) finding that beta-band responses are involved in rhythmic beat processing is interesting in light of Zatorre et al.'s (2007) and Grahn and Rowe's (2009) suggestion that auditory-motor interactions underlie the temporal predictions involved in auditory rhythm perception. Beta frequencies have been intimately associated with the motor system, and a recent hypothesis suggests that they are also associated with endogenously-driven top-down cognitive processes (Engel and Fries, 2010). Furthermore, as noted by Iversen et al. (2009), beta band activity has been theoretically shown to be able to mediate longer-distance cortical coupling than gamma band activity, suggesting that beta-band activity could reflect functional coupling of distant brain regions, such as auditory and motor planning regions (cf. Bartolo et al., 2014 for relevant neural data).

**Figure 3:**
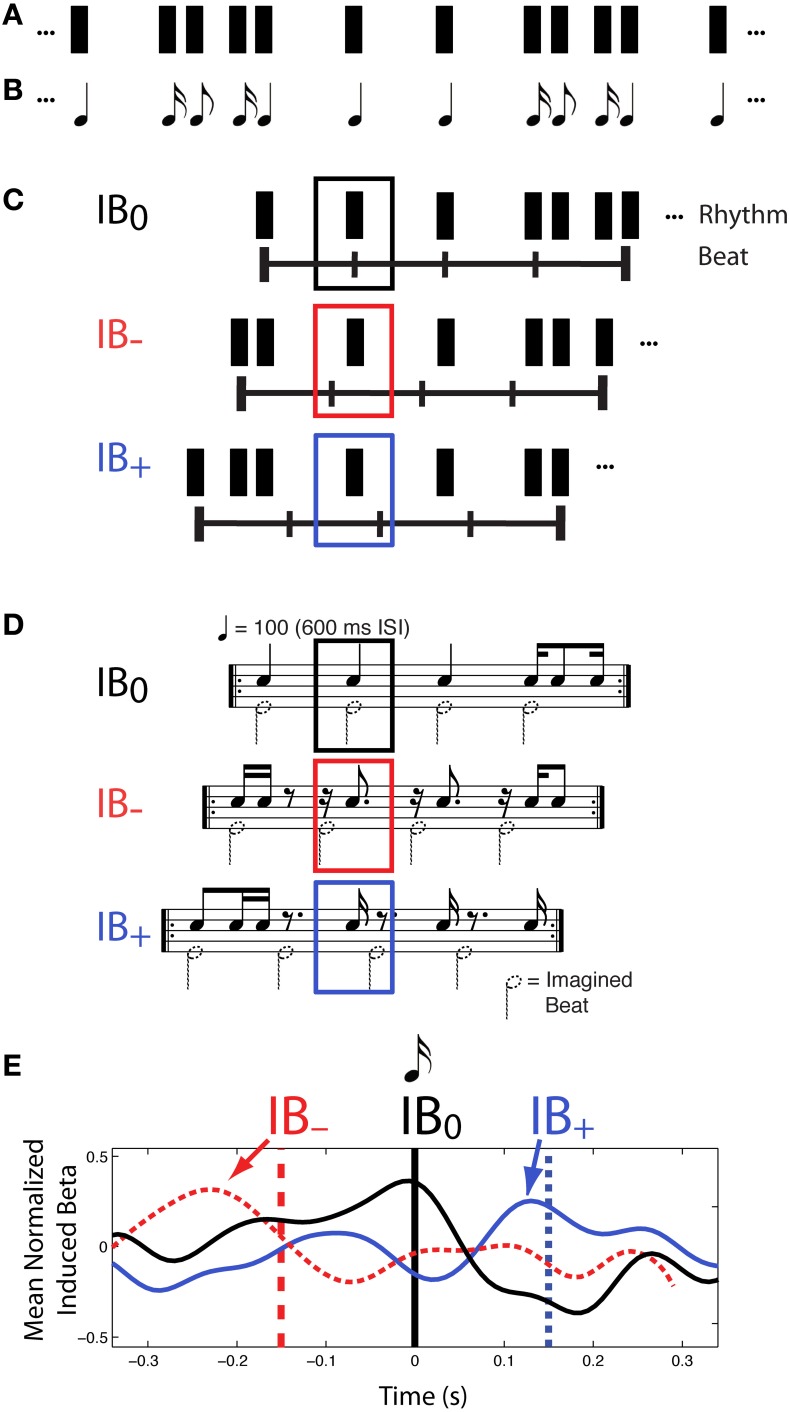
**Patterns of induced beta-band neural activity as a function of imagined beat location in a syncopated rhythm, adapted from Iversen et al. (2009)**. **(A)** Constant rhythmic pattern where individual notes are indicated by black rectangles. **(B)** Music notation of the same rhythm pattern. **(C)** On different trials, participants mentally organized the perceived beat structure of a syncopated rhythm so that all beats fell on sounded tones (condition IB0) or some beats fell on silent positions just before (IB−) or after (IB+) sounded tones, (IB = Imagined Beat). The horizontal line with vertical tick marks indicates the timing of the imagined beats, and the rectangles above indicate the repeating rhythmic unit for each beat organization. **(D)** Music notation of three beat structures and associated rhythms shown in **(C)**. Black notes show sounded tones, while notes with dotted note heads show imagined beats. The sounded-tone pattern is physically identical in all three conditions, but is psychologically distinct depending on where one feels the beat. In both **(C,D)** black, red and blue squares indicate the analysis window for MEG data, which is centered on the same tone. **(E)** Patterns of induced beta-band neural activity for beats on actual tones (marked by the vertical black line at 0 ms) vs. beats on silent positions just before (red) or after (blue) sounded tones. For the beat-at-silent-position conditions, the vertical dashed lines show the location of the imagined beats, relative to the sounded tone at time 0, and the grand mean normalized fluctuation of induced beta-band activity shows a peak of power that reflects the timing of the imagined beat (arrows), not the auditory input. Note how in all three conditions, the power of the induced beta-band signal rises in anticipation of the time of the beat, and sharply decreases around the time of the beat. Beta-band fluctuation was computed by subtracting the mean over the entire interval (−300 to 300 ms).

Further evidence for a role of beta frequencies in beat processing, and specifically in the prediction of beat times, comes from another experiment reported in Iversen et al. (2009). Again, a constant physical sound pattern was presented to listeners (Figures 3A,B), who were asked to hear the beat in different positions in the rhythm on different experimental trials, yielding highly distinct rhythmic percepts. This time, however, syncopated rhythms were used so that on some trials listeners felt a beat at locations where there was no sound (Figures 3C,D, conditions IB− and IB+). This approach allows stimulus-driven auditory neural activity to be dissociated from endogenous, beat-related activity.

In this study Iversen et al. separately examined evoked and induced beta-band responses. Evoked neural responses are phase-locked to a stimulus, while induced responses are not (Tallon-Baudry and Bertrand, 1999). In previous neural research on rhythm perception, Snyder and Large (2005) had found that induced (but not evoked) beta and gamma-band neural activity anticipated the timing of upcoming tones in an isochronous rhythm, and had suggested that brain oscillations could be a neural signature of rhythmic expectancy (cf. Large and Snyder, 2009). Iversen et al.', (2009) study built on this work, but the approach differed from that of Snyder and Large (2005) in using voluntary control of beat perception in syncopated rhythms, rather than occasional omission of tones in an isochronous sequence, to probe the neural correlates of beat perception. A primary motivation for Iversen et al.', (2009) approach was that it allows one to disentangle “cognitive” and “sensory” expectations. When sounds are occasionally omitted at beat positions in a repeating pattern, any expectancy-related signals partly reflect the brain's expectation for sensory input at that point. In contrast, by using syncopated rhythms in which perceived beats occur at points where sound *never* occurs, one can examine neural correlates of beat perception driven purely by cognitive representations, rather than by a combination of cognitive and sensory expectations. Using this approach, Iversen et al. (2009) found that while evoked beta-band responses tracked physical sound onsets, *induced* beta-band responses tracked the location of imagined beats. Notably, the peak and following decline of induced beta-band activity slightly anticipated or coincided with the time of the imagined beat (Figure 3E), even when the beat occurred at a silent position in the syncopated rhythm. This suggested that modulation of induced beta-band activity represents beat-related processes, possibly including predictions of upcoming beats.

Recently Fujioka et al. (2012) also explored the role of beta-band oscillations in purely perceptual beat processing using MEG. They compared isochronous tone sequences (with inter-onset-intervals of 390, 585, or 780 ms) to sequences in which tone onsets occurred at random temporal intervals. Unlike the study of Iversen et al. (2009), in which attention was directed toward the beat structure of the stimuli, in Fujioka et al.'s study, participants were told to “pay no particular attention to the sound” and watched a silent movie, under the assumption that beat processing is an automatic response to rhythmic stimuli (though see Chapin et al., 2010, for possible problems with this assumption). In all four conditions the researchers found a sharp decrease in beta-band power soon after each tone onset (beta desynchronization). However, in the isochronous conditions they observed a gradual build-up in beta-band power before the onset of the following tone, such that the amplitude of this signal peaked just before tone onset. Fujioka et al. (2012) suggest that this beta-band activity may reflect a neural mechanism for predicting the timing of beats. Furthermore, source localization and phase coherence measures indicated that there were temporally correlated beta-band modulations in auditory regions and motor planning regions (including the supplementary motor area). This suggests that neural oscillations in the beta range may reflect functional coupling between these regions.

In summary, existing MEG and EEG research provides data consistent with the ASAP hypothesis: beat perception appears to involve precisely-timed modulation of auditory neural activity around the time of perceived beats. Furthermore, the nature of this activity (specifically, the involvement of beta-band oscillations and correlated modulations between beta-band activity in auditory and motor planning regions) implies that the motor system plays a role in producing these modulations.

### Three predictions of the ASAP hypothesis

It is possible, of course, that all of the above neural findings could be explained by a “Hebbian” (fire-together wire-together) view of learned connections between the auditory and motor systems in beat perception. That is, since humans frequently move to the beat of music, beat-related processing in the auditory system (even in the absence of movement) may co-activate motor planning regions simply due to the frequent temporally-correlated activity in auditory and motor regions during actual movement to music. Should this be the case, then motor system activations during rhythmic beat perception would be a case of “corollary firing” without any functional significance.

One of the key features of ASAP is that it adopts a different view, namely that the motor system plays a *causal* role in beat perception. This leads to testable predictions not made by the Hebbian view (or, to our knowledge, by neural resonance theory). Specifically, ASAP predicts that the disruption of normal activity in motor planning regions will impair beat perception. For example, Chen et al. (2008a) found that mid premotor cortex (midPMC) was active when listeners perceived musical rhythms with a beat, even though the listeners were not moving or anticipating that movement would be required. Accordingly, ASAP predicts that if midPMC is transiently deactivated using transcranial magnetic stimulation (TMS), then performance on purely perceptual tests of musical beat processing (such as the BAT, Iversen and Patel, 2008), will also be impaired. TMS of premotor cortex has been used to study motor synchronization with rhythmic patterns (Kornysheva and Schubotz, 2011), but to our knowledge has not been used to study beat processing in a purely perceptual paradigm. A recent TMS study by Stupacher et al. (2013) provided intriguing evidence that beat perception modulates motor system excitability, but used TMS over motor rather than premotor cortex, and did not measure beat perception directly.

A second prediction of ASAP concerns interference experiments. If motor planning activity for periodic movements is involved in predicting the timing of musical beats, then if people are asked to perceive musical beats while their motor planning system is occupied with producing or planning non-beat-related movements, this should interfere with beat perception. One way of testing this idea is to have individuals do purely perceptual tests of beat perception (e.g., the BAT) while moving their arms and legs rhythmically at a tempo unrelated to the musical beat (for example, using an “elliptical” training machine), or while doing a demanding non-beat-based visuomotor tracking task. ASAP predicts that this should disrupt performance on beat perception tests. Such movement, however, should not disrupt equally-difficult music perception tasks that do not engage beat perception/motor planning regions, such as same/different discrimination of short melodic sequences. Studies which attempt to disrupt beat perception with non-beat-related periodic movements would complement recent research showing that moving in synchrony with an auditory beat *enhances* the perception of beat timing (Manning and Schutz, 2013).

A third prediction of ASAP concerns neuroimaging research. As discussed earlier, a growing number of fMRI studies indicate that pure beat perception involves motor system activation as well as functional coupling between motor planning regions and auditory regions. Such studies, however, have not addressed the direction of signal flow between auditory and motor regions. ASAP posits precise, two-way neural signaling between these regions, with motor-to-auditory signals playing a causal role in beat perception (specifically, in supporting temporal predictions for upcoming beats). Thus, ASAP predicts that future neuroimaging work which allows the measurement of directional patterns in neural signals will reveal that motor-to-auditory signals play an important role in beat perception. Testing this idea will likely require a combination of neuroimaging methods sensitive to temporal and spatial patterns of brain activity, such as MEG and fMRI, as well as techniques for describing directed information flow between brain regions (e.g., Brookes et al., 2011).

## Neural substrates for auditory-motor interactions in beat perception: an evolutionary perspective

### The dorsal auditory pathway as a possible substrate

What connections in the human brain might support the types of neural interactions posited by the ASAP hypothesis? Any such connections need to satisfy three criteria. First, they must link auditory regions and motor planning regions, with the latter including regions for body (non-orofacial) movements, since the movement simulations involved in ASAP likely involve trunk, head, and limbs movements, based on how people actually move to music (Burger et al., 2013). Second, the connections must support temporally precise two-way signaling between auditory regions and motor planning regions. Third, the connections should be much more developed in humans than in other primates, to account for human-monkey differences in beat perception and synchronized tapping to a metronome (Zarco et al., 2009; Honing et al., 2012; cf. the Introduction).

**Figure 4:**
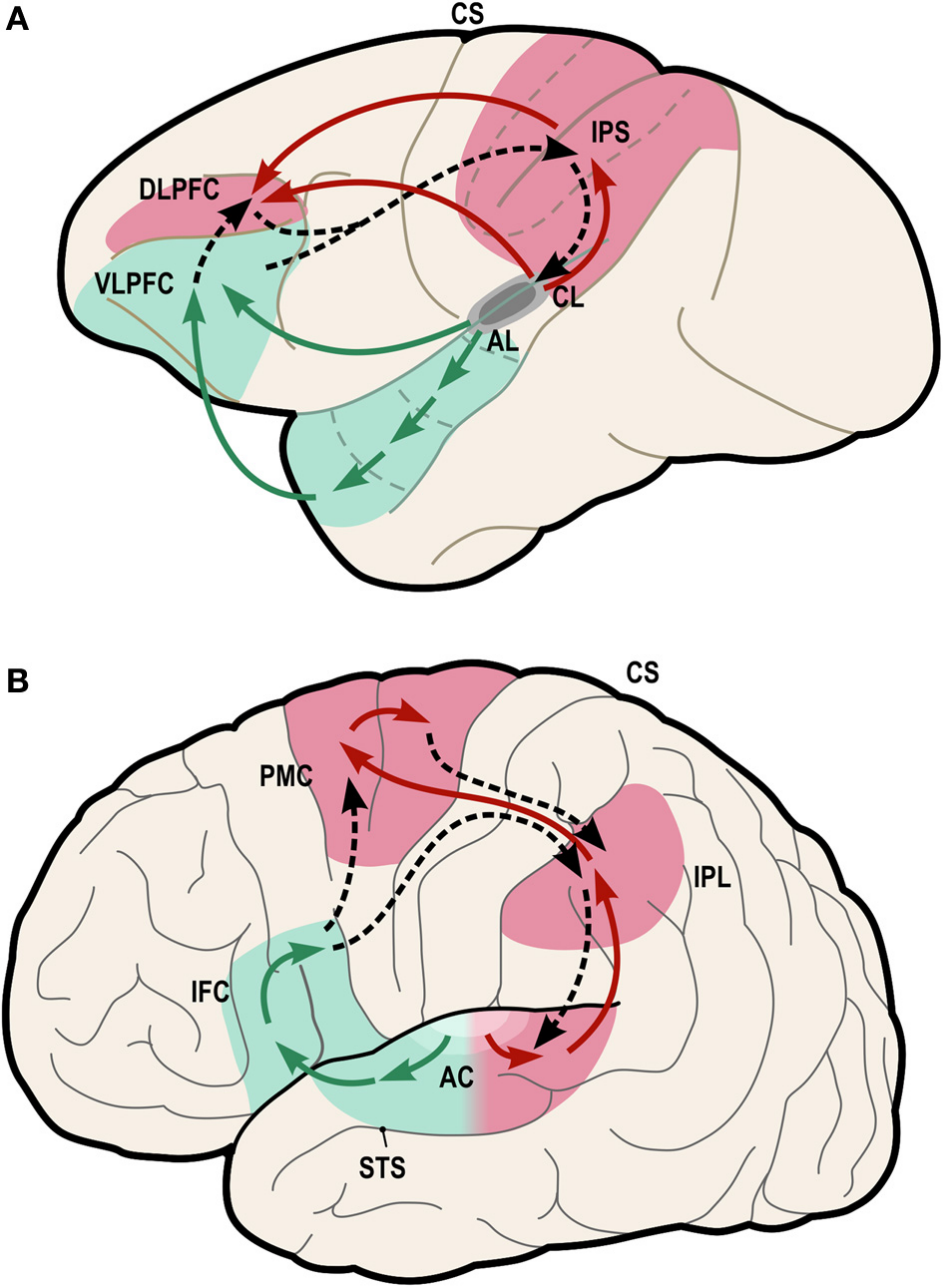
**Model of dual-stream auditory processing in the primate brain, from Rauschecker (2011)**. Dorsal (red) and ventral (green) auditory pathways are shown in the macaque brain **(A)** and the human brain **(B)**. Solid arrows indicate ascending projections from auditory cortex, while dashed arrows indicate reciprocal projections back to the auditory cortex. AC, auditory cortex; AL/CL, anterolateral/caudolateral superior temporal gyrus; CS, central sulcus; DLPFC, dorsolateral prefrontal cortex; IFC, inferior frontal cortex; IPL, inferior parietal lobule; IPS, inferior parietal sulcus; PFC, prefrontal cortex; PMC, premotor cortex; STS, superior temporal sulcus; VLPFC, ventrolateral prefrontal cortex.

One possible neural pathway that could satisfy these criteria is the “dorsal auditory pathway” (or “dorsal stream” pathway), which links caudal auditory regions with dorsal frontal premotor regions via parietal regions (Figure 4, red regions).

Rauschecker and Tian (2000) first proposed that this pathway and the ventral auditory pathway (Figure 4, green regions) play distinct and complementary roles in auditory processing, with the former subserving localization of sounds in space and the latter subserving identification of “auditory objects,” including speech sounds. The dorsal stream has also been proposed to play a role in speech processing, especially phonological processing and sensorimotor control (Hickok and Poeppel, 2007). Rauschecker and Scott (2009) have suggested that this pathway provides certain computational capacities important for both spatial and speech processing, as “both share a common set of properties that actually require a neural system like the dorsal stream, which creates an interface between sensory and motor networks and performs a matching operation between predicted outcomes and actual events.” (Rauschecker, 2011). Germane to the ASAP hypothesis, Rauschecker (2011) notes that this expanded view of the dorsal stream “transforms it from a purely sensory or afferent pathway into an equally efferent pathway, in which predictive motor signals modify activity in sensory structures.” Also of interest from the standpoint of the ASAP hypothesis, there is evidence that parietal cortex plays a role in auditory temporal processing in humans (Foster et al., 2013).

**Figure 5:**
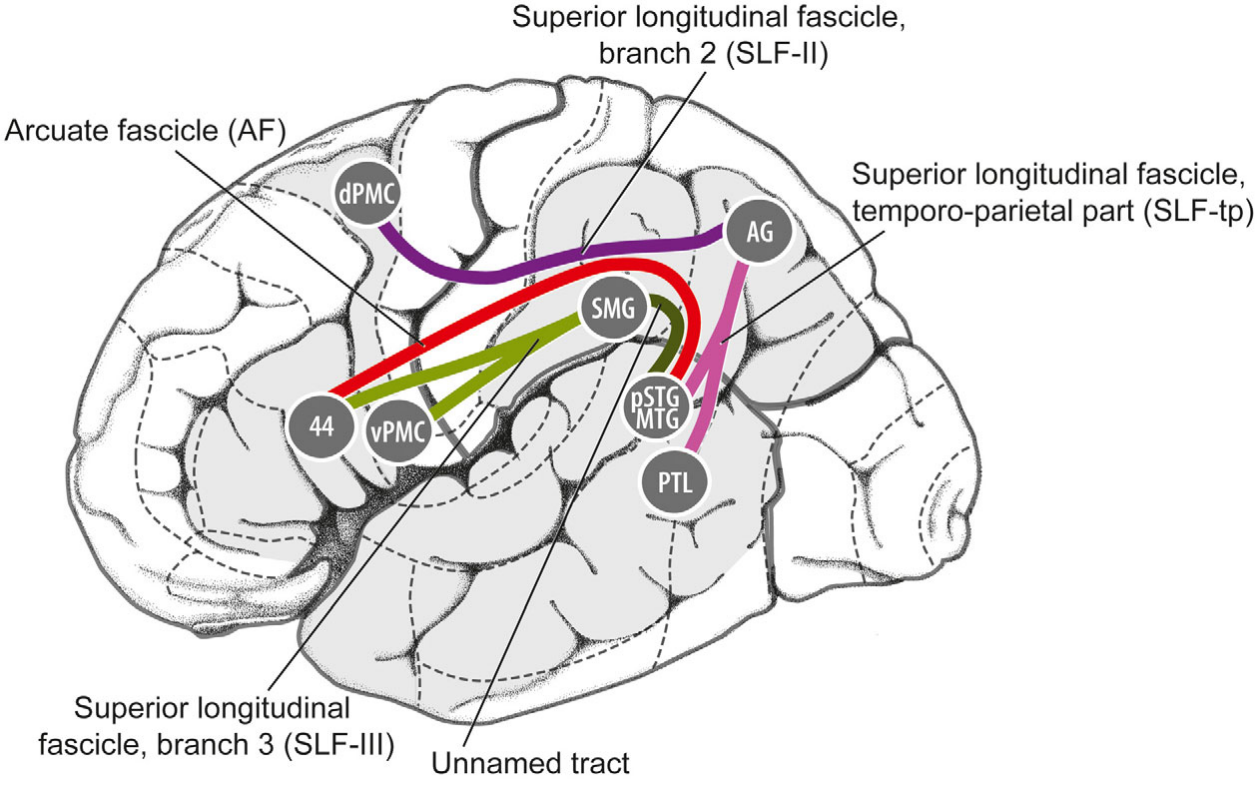
**Details of some of the fiber tracts thought to be involved in the dorsal auditory processing stream in humans, from Gierhan (2013)**. 44, Brodman Area 44; AG, angular gyrus; dPMC, dorsal premotor cortex; pSTG/MTG, posterior superior temporal gyrus/middle temporal gyrus; PTL, posterior temporal lobe; SMG, supramarginal gyrus; vPMC, ventral premotor cortex.

Anatomically, it appears that this pathway could satisfy the first criterion mentioned above, i.e., linking auditory regions and motor planning regions for non-orofacial movements. In particular, as shown in Figure 5, the pathway from caudal auditory cortex [pSTG, posterior superior temporal gyrus] to the parietal lobe [AG, angular gyrus] via the temporo-parietal division of the superior longitudinal fasciculus [SLF-tp], and the pathway from the parietal cortex to the dorsal premotor cortex [dPMC] via the 2nd branch of the superior longitudinal fasciculus [SLF II] could provide a route for signals to be exchanged between caudal auditory cortex and dorsal premotor cortex in humans. Germane to the ASAP hypothesis, dorsal premotor regions are involved in motor planning of trunk and limb movements (ventral premotor regions are more heavily involved in control of orofacial movements). Furthermore, regions in this dorsal pathway have rich connections with the basal ganglia/putamen, another brain structure known to be important in beat perception (Grahn and Rowe, 2009; Teki et al., 2012; Kung et al., 2013; Merchant and Honing, 2014).

Turning to the second criterion, can the dorsal auditory pathway support temporally-precise two-way signaling between motor planning regions and auditory regions? It is notable that auditory neurons in caudal auditory fields (which would be part of the dorsal processing stream) have significantly shorter neural response latencies than those in rostral fields (Camalier et al., 2012; cf. Kuśmierek and Rauschecker, 2014), which would favor their involvement in temporally-precise interactions with motor planning regions. Furthermore, there is evidence for two-way signal flow between premotor and auditory regions in humans. An MEG study of the suppression of auditory activity by silent lipreading by Kauramäki et al. (2010) suggested that efference copies of neural activity from motor planning regions can influence auditory cortical activity, while an fMRI study of phoneme categorization by Chevillet et al. (2013) suggested that signals travel from posterior superior temporal gyrus to premotor cortex during perceptual tasks.

Finally, the dorsal auditory pathway might be a possible locus of neuroanatomical differences between humans and monkeys. Both monkeys and humans have a well-developed dorsal auditory stream (Romanski et al., 1999; Hackett, 2011; Rauschecker, 2011), but the details of neuroanatomical connectivity may differ in ways pertinent to the ASAP hypothesis. In particular, the pathway linking caudal auditory regions to dorsal premotor regions via the parietal cortex may differ between humans and monkeys. More specifically, while the neuroanatomy of SLF II (which connects parietal and premotor cortex) appears to fairly similar in humans and monkeys (de Schotten et al., 2012), there may be significant species differences in the strength of the connections between superior temporal regions and parietal regions (i.e., the SLF-tp connections in Figure 5; cf. the “posterior indirect segment” of the arcuate fasciculus in Figure 2 of López-Barroso et al., 2013). These latter connections may be much stronger in humans than in monkeys, perhaps due to the evolution of vocal learning in our lineage (i.e., our ability to mimic a wide range of sounds, a capacity lacking in monkeys; cf. Warren et al., 2005; Rilling et al., 2008). Pertinent to this point, in a neuroanatomical study Lewis and Van Essen (2000) found that projections from the caudal belt of the STG to the posterior parietal cortex in macaque monkeys were rather sparse (Lewis and Van Essen, 2000), suggesting that the strength of these projections may be an important neuroanatomical difference between humans and monkeys. Species differences in the pathways linking temporal and premotor cortex have also recently been proposed by Merchant and Honing (2014) to explain why synchronization with periodic stimuli is generally inferior in monkeys when compared to humans.

Before closing this section, it is worth addressing how the dorsal auditory stream relates to neural pathways involved in the mirror neuron system, since both have been proposed as substrates for sensorimotor integration (Rizzolatti and Sinigaglia, 2010). For example, Kohler et al. (2002) found neurons in the frontal cortex of macaque monkeys that responded when a monkey performed a hand action (such as tearing a piece of paper), or when they heard the sound of the same action being performed out of sight of the monkey. Crucially, however, these neurons were found in a *ventral* premotor area (area F5, see Rizzolatti and Sinigaglia, 2010, Figure 1), which is anatomically distinct from the mid and dorsal premotor areas implicated in beat perception and synchronization of movement to a beat (Chen et al., 2008a,b). Thus, the mirror neuron system is likely to be distinct from neural connections of interest here, i.e., pSTG-AG-dPMC (Figure 5). Those interested in possible roles the mirror system may play in music processing may consult (Koelsch, 2012, Ch. 11) for a brief review.

### Cross-species research on beat perception: existing research and future directions

Studying beat perception in other species is essential for discovering if this capacity is widespread (as implied by Darwin's view of rhythm and by neural resonance theory) or if it species-restricted. The finding that rhesus monkeys do not seem to perceive a beat in rhythmic sequences (Honing et al., 2012) raises the possibility that the capacity is species-restricted, though more behavioral and neural work is needed to see if non-human primates truly lack the capacity to perceive a beat (cf. Geiser et al., 2014).

In thinking about cross-species research on beat perception, it is important to be precise about what is meant by “perceive a beat.” As reviewed earlier in this paper, musical beat perception in humans has several key aspects. In terms of comparison to other species, where the main observable behavior is motor synchronization to a beat, the two most important aspects are (1) the predictive nature of beat perception and (2) the flexibility of beat perception across a wide range of tempi. Humans demonstrate these aspects when they move in synchrony with the beat of music: their movements are closely aligned in time with beats (indicating accurate temporal prediction), and they can do this at a wide range of musical tempi (indicating tempo flexibility).

Recently, this sort of predictive and tempo-flexible synchronization to a musical beat has been demonstrated in a few species of non-human animals, including several parrot species, the Asian elephant, and the California sea lion (Patel et al., 2009; Schachner et al., 2009; Hasegawa et al., 2011; Cook et al., 2013). The parrots and Asian elephant are known vocal learners (Fitch, 2013), consistent with the “vocal learning and rhythmic synchronization hypothesis” (Patel, 2006), which posits that neural changes in auditory-motor circuitry driven by the evolution of vocal learning laid the foundation for the capacity to synchronize movement to the beat of music. The vocal learning hypothesis entails the idea that the evolution of vocal learning led to more general integration of auditory and motor regions of the brain than just the circuits connecting auditory and vocal motor control centers (cf. Petkov and Jarvis, 2012).

Sea lions, however, are not known to be vocal learners, which challenges the vocal learning hypothesis. However, it may be premature to argue that this refutes the hypothesis. This is because sea lions are related to true seals and to walruses, which are known vocal learners (Arnason et al., 2006; Schusterman, 2008). Hence the absence of evidence for vocal learning in sea lions is not strong evidence of absence of this capacity or its underlying neural mechanisms. To test whether California sea lions are really vocally inflexible, behavioral training studies of vocal flexibility in this species are needed, particularly since the most recent experimental studies of sea lion vocal flexibility date from the 1960s and 1970s (Schusterman, 2008). Structural neuroimaging of sea lions brains using diffusion tensor imaging (DTI) would also be of interest, to study auditory-premotor connections in the dorsal auditory pathway (and in particular, the pSTG-AG-dPMC pathway shown in Figure 5). It may be, for example, that sea lions retain strong dorsal pathway premotor-auditory connections inherited from a vocal-learning common ancestor of true seals, sea lions, and walruses (cf. Patel, 2014).

**Figure 6:**
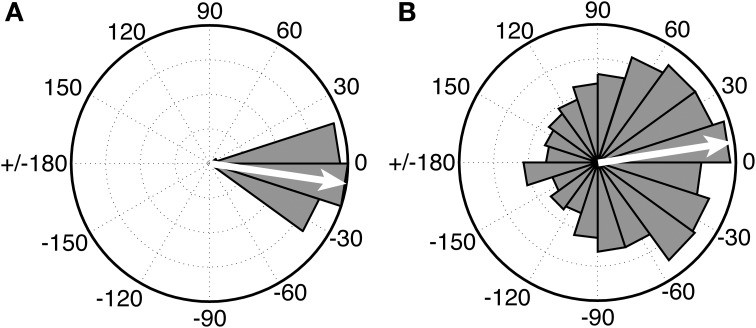
**Circular histograms of relative phase values for human (A) vs. chimpanzee (B) taps to an auditory metronome with a period of 600 ms**. In these plots asynchronies between taps and tones are expressed as relative phase values: 0° indicates taps perfectly aligned with tones, 180° indicates taps midway between tones, negative values (e.g., −15°) indicate taps before tones, and positive values (e.g., 15°) indicate taps after tones. Human data (Left) from Iversen et al., in press. Chimpanzee data (Right) redrawn from Hattori et al. (2013). In both graphs, the mean relative phase angle is shown with an inset arrow.

Of substantial interest for future comparative work on beat perception is research with chimpanzees, who are our closest living primate relatives, and who are known to drum in the wild at part of their natural display behavior (Fitch, 2006). While there is no purely perceptual research on beat processing in apes, the first study of synchronization to an auditory metronome in apes was recently published (Hattori et al., 2013). In this study three chimpanzees (*Pan troglodytes*) were tested for synchronization to a metronome at three different tempi. One chimp (named “Ai”) synchronized her taps to the metronome at one tempo (period = 600 ms), which was close to her spontaneous tapping tempo. However, she did not synchronize at the other two metronome tempi, and the other two chimps did not sync their taps to the metronome at any tempo. Thus, no chimpanzee showed tempo flexibility in synchronization. Even for Ai's synchronized tapping, there were notable differences from human synchronization to a metronome. Figure 6 (left) shows a circular histogram of relative phase values between taps and tones for a human adult tapping to an auditory metronome with a period of a 600 ms (from Iversen et al., in press). The relative phase values cluster tightly around 0 degrees (which corresponds to perfect alignment between taps and tones). Figure 6 (right), from Hattori et al. (2013), shows the relative phase values of Ai's tapping to a metronome with a period of 600 ms.

Although the mean relative phase of Ai's taps is close to 0, the distribution of her relative phase values is much wider, indicating that many of her taps were distant from metronome tones in time. Thus, further work is needed to see if her performance improves with practice, or if data like that in Figure 6 (right) represent the best a chimp can do, which would suggest rather imprecise prediction of beat timing^1^. For the current purposes, however, the key point is that there is at present no evidence for beat perception in chimps or any other non-human primate that is both predictive and tempo-flexible.

While testing synchronization to a beat is one way to test beat perception in other species, in future studies it will also be important to conduct purely perceptual tests, since an animal may be able to perceive a beat without being able to synchronize movements with it. The key issue is whether the induction of a beat shapes the perceptual processing of rhythm, e.g., attentional selection (Large and Jones, 1999), accent perception, and grouping of rhythmic patterns. Recently, a purely perceptual test of rhythm processing in monkeys demonstrated that the animals were sensitive to changes in a repeating temporal pattern (Selezneva et al., 2013). In that study it was not possible to tell if the monkeys were reacting to a change in the perceived grouping of an auditory pattern or to a change in the underlying beat pattern, and thus further such research is needed. In addition to behavioral methods one could also look for neural correlates of beat perception in other species, e.g., brain oscillations that show peaks in power just prior to beat times (as in Iversen et al., 2009; Fujioka et al., 2012; cf. Jaramillo and Zador, 2011), or which reflect beat frequency (Nozaradan et al., 2012). However, purely neural data in the absence of any behavioral evidence of beat perception must be interpreted with caution. As shown by Bidelman et al. (2011), Moreau et al. (2013), and others, neural signals associated with auditory processing do not always indicate perceptual abilities. Thus, future animal neuroscience work examining beat perception will need to combine neural and behavioral measures. One idea for behavioral measures comes from research on humans which shows facilitated perceptual processing of auditory events which occur on (vs. off) the beat (Escoffier et al., 2010; Geiser et al., 2012). Such paradigms could be adapted for research on non-human animals. If an animal shows perceptual facilitation for events that occur on the beat, and this facilitation can be demonstrated at a broad range of different tempi, this would suggest that the animal perceives the beat.

## Conclusion

This paper argues that the neural mechanisms of musical beat perception involve action simulation in the service of auditory prediction, as well as temporally-precise two-way interactions between motor planning regions and auditory regions of the brain. That is, we hypothesize that musical beat perception depends on strong functional connections between motor and auditory regions by which motor planning signals can fundamentally influence auditory processing and perception. This “action simulation for auditory prediction” (ASAP) hypothesis leads to several testable predictions. This paper also suggests that the neural substrates of beat perception involve the dorsal auditory pathway, and that this pathway may differ between humans and other primates due to the evolution of vocal learning in our lineage. If this proves to be the case, then beat perception, far from being a widespread capacity among animals, may be surprisingly rare in the animal kingdom.

### Conflict of interest statement

The authors declare that the research was conducted in the absence of any commercial or financial relationships that could be construed as a potential conflict of interest.

## Footnotes

^1^One reason that the relative phase values are so much more widely spread in the chimpanzee data may be due to the method used to study chimpanzee tapping. As described by these authors, “we introduced an electric keyboard to three chimpanzees and trained them to tap two keys (i.e., ‘C4’ and ‘C5’) alternately 30 times…. Each key to be tapped was illuminated, and if a chimpanzee tapped this key (e.g., ‘C4’), sound feedback was given and another key was immediately illuminated (e.g., ‘C5’) so it was unlikely that the visual stimuli affected tapping rhythm by chimpanzees. When the chimpanzees tapped the two keys in alternation a total of 30 times, they received positive auditory feedback (a chime) and a reward.” Once this task was learned, the experimenters introduced a background metronome sound to see if the chimps would spontaneously align their taps with that sound. While this is a clever paradigm, it focuses the animal's attention on the lighted keyboard rather than on the background sound, which is task-irrelevant. The results may thus underestimate the abilities of chimpanzees to synchronize to a metronome. Finding a way to make synchronizing with the background sound more task-relevant seems an important direction for this research.
